# Are Heavy Fermion Strange Metals Planckian?

**DOI:** 10.3390/cryst12020251

**Published:** 2022-02-12

**Authors:** Mathieu Taupin, Silke Paschen

**Affiliations:** Institute of Solid State Physics, Vienna University of Technology, Wiedner Hauptstr. 8-10, 1040 Vienna, Austria; taupin@ifp.tuwien.ac.at

**Keywords:** heavy fermion compounds, strange metals, Planckian dissipation, quantum criticality, Kondo destruction

## Abstract

Strange metal behavior refers to a linear temperature dependence of the electrical resistivity that is not due to electron–phonon scattering. It is seen in numerous strongly correlated electron systems, from the heavy fermion compounds, via transition metal oxides and iron pnictides, to magic angle twisted bi-layer graphene, frequently in connection with unconventional or “high temperature” superconductivity. To achieve a unified understanding of these phenomena across the different materials classes is a central open problem in condensed matter physics. Tests whether the linear-in-temperature law might be dictated by Planckian dissipation—scattering with the rate $∼k_{B}T/\hbar$—are receiving considerable attention. Here we assess the situation for strange metal heavy fermion compounds. They allow to probe the regime of extreme correlation strength, with effective mass or Fermi velocity renormalizations in excess of three orders of magnitude. Adopting the same procedure as done in previous studies, i.e., assuming a simple Drude conductivity with the above scattering rate, we find that for these strongly renormalized quasiparticles, scattering is much weaker than Planckian, implying that the linear temperature dependence should be due to other effects. We discuss implications of this finding and point to directions for further work.

## 1. Introduction

A first step in understanding matter is to delineate the different phases in which it manifests. To do so, a characteristic that uniquely identifies a phase must be found, and using its order has worked a long way. How this classification should be extended to also incorporate topological phases [1] is a matter of current research. Here, we focus on topologically trivial matter and thus take order-parameter descriptions [2] as a starting point and consider the case of second-order phase transitions. As an order parameter develops below a transition (or critical) temperature, the system’s symmetry is lowered (or broken). Cornerstones are the power law behavior of physical properties near the critical temperature, with universal critical exponents, and the associated scaling relationships. Combined with renormalization-group ideas [3], this framework is now referred to as the Landau–Ginzburg–Wilson (LGW) paradigm. It has also been extended to zero temperature. Here, phase transitions—now called *quantum* phase transitions [4]—can occur as the balance between competing interactions is tipped. To account for the inherently dynamical nature of the T=0 case, a dynamical critical exponent needs to be added. This increases the effective dimensionality of the system, which may then surpass the upper critical dimension for the transition, so that the system behaves as noninteracting, or “Gaussian”. Interestingly, however, cases have been identified where this expectation is violated [5,6,7,8], evidenced for instance by the observation of dynamical scaling relationships [9] that should be absent according to the above rationale. We will refer to this phenomenon as “beyond order parameter” quantum criticality. It appears to be governed by new degrees of freedom specific to the quantum critical point (QCP). This is a topic of broad interest both in condensed matter physics and beyond, but a general framework is lacking. We will here discuss it from the perspective of heavy fermion compounds, where it can manifest as Kondo destruction quantum criticality [5,6]. We will in particular discuss materials that display linear-in-temperature “strange metal” electrical resistivity, as well as the proposed relation [10,11] to Planckian dissipation. We will allude to similar phenomena in other material platforms and point to directions for further research to advance the field.

## 2. Simple Models for Strongly Correlated Electron Systems

Strongly correlated electron systems host electrons at the brink of localization. The simplest model that can capture this physics is the Hubbard model

(1) $$H=-t\sum_{\langle ij\rangle ,\sigma }\left(d_{i\sigma }^{†}d_{j\sigma }+d_{j\sigma }^{†}d_{i\sigma }\right)+U\sum_{i}d_{i↑}^{†}d_{i↑}d_{i↓}^{†}d_{i↓}\;.$$

The hopping integral *t* transfers electrons from site to site and thus promotes itineracy, whereas the onsite Coulomb repulsion *U* penalizes double occupancy of any site, thereby promoting localization. Thus, with increasing U/t, a (Mott) metal–insulator transition is expected. This simple model is suitable for materials where transport is dominated by one type of orbital with moderate nearest neighbor overlap, leading to one relatively narrow band. Well-known examples are found in transition metal oxides, for instance the cuprates. Here, the relevant orbitals are copper *d* orbitals, kept at distance by oxygen atoms. The creation and annihilation operators are called *d* and $d^{†}$ here.

If two different types of orbitals interplay—one much more localized than the other—a better starting point for a theoretical description is the (periodic) Anderson model that, for the one-dimensional case, reads [12,13]

(2) $$H=\sum_{k,\sigma }\epsilon_{k}c_{k\sigma }^{†}c_{k\sigma }+\sum_{j,\sigma }\epsilon_{f}f_{j\sigma }^{†}f_{j\sigma }+U\sum_{j}f_{j↑}^{†}f_{j↑}f_{j↓}^{†}f_{j↓}+\sum_{j,k,\sigma }V_{jk}\left(e^{ikx_{j}}f_{j\sigma }^{†}c_{k\sigma }+e^{-ikx_{j}}c_{k\sigma }^{†}f_{j\sigma }\right)\;.$$

Orbitals with large overlap, with the associated creation and annihilation operators *c* and $c^{†}$, form a conduction band with dispersion $\epsilon_{k}$. Orbitals with vanishing overlap situated at the positions $x_{j}$ are associated with the operators *f* and $f^{†}$. They are assumed to be separated by a distance greater than the *f* orbital diameter and thus no hopping between them is considered. However, the hybridization term *V* allows the *f* electrons to interact. This model is particularly well suited for the heavy fermion compounds, which contain lanthanide (with partially filled 4f shells) or actinide elements (with partially filled 5f shells) in addition to *s*, *p*, and *d* electrons. For the so-called Kondo regime, where *f* orbitals effectively act as local moments, the Anderson model can be transformed into the Kondo (lattice) model

(3) $$H=\sum_{k,\sigma }\epsilon_{k}c_{k\sigma }^{†}c_{k\sigma }-J\sum_{i}{\vec{S}}_{i}\cdot c_{i,\sigma }^{†}{\vec{\sigma }}_{\sigma ,\sigma^{\prime }}c_{i\sigma^{\prime }}\;,$$

where the interaction between the localized and itinerant electrons is expressed in terms of an antiferromagnetic exchange coupling *J*. $\vec{S}$ is the local magnetic moment of the *f* orbital and ${\vec{\sigma }}_{\sigma ,\sigma^{\prime }}$ are the Pauli spin matrices. One of the possible ground states of this model is a paramagnetic heavy Fermi liquid with a large Fermi surface, which contains both the local moment and the conduction electrons. The resonant elastic scattering at each site generates a renormalized band at the Fermi energy. Its width is of the order of the Kondo temperature $T_{K}$, which can be orders of magnitude smaller than the noninteracting band width. In the (typically considered) simplest case (with a uniform and *k* independent hybridization), this band extends across essentially the entire Brillouin zone.

In popular terms, this heavy fermion band could be seen as the realization of a nearly perfect “flat band” (an early description of an interaction-driven truly flat band, with zero energy, is given in [14] and its relevance for strange metal physics is discussed in [15,16]). Flat bands have also been predicted [17] and later identified in magic angle twisted bi-layer graphene (MATBG) [18] as a result of moiré band formation, and are expected in lattices with specific geometries [19,20] such as the kagome lattice [21,22] through destructive phase interference of certain hopping paths. Whereas the theoretical description of these latter flat band systems may be simpler than solving even the simplest Hamiltonians for strongly correlated electron systems, such as (1)–(3), the inverse might be true for the challenge on the experimental side. Heavy fermion compounds with a large variety of chemical compositions and structures [23,24,25] can be quite readily synthesized as high-quality (bulk) single crystals; the heavy fermion “flat bands” are robust (not fine tuned), naturally extend essentially across the entire Brillouin zone, and are pinned to the Fermi energy. Albeit, they form in the Kondo coherent ground state of the system, which is typically only fully developed at low temperatures. To realize such physics via a complementary route that might bring these properties to room temperature is an exciting perspective. Bringing together these different approaches bears enormous potential for progress. Indeed, for both twisted trilayer graphene [26] and MATBG [27] the connection to heavy fermion physics has very recently been pointed out. Another topic discussed across the various platforms is “strange metal” physics, which we address next.

## 3. Strange Metal Phase Diagrams

Metals usually obey Fermi liquid theory, even in the limit of strong interactions. This is impressively demonstrated by the large body of heavy fermion compounds that, at sufficiently low temperatures, display the canonical Fermi liquid forms of the electronic specific heat

(4) $$C_{p}=\gamma T\;,$$

the Pauli susceptibility

(5) $$\chi =\chi_{0}\;,$$

and the electrical resistivity

(6) $$\rho =\rho_{0}+AT^{2}\;,$$

where $\rho_{0}$ is the residual (elastic) resistivity. Theoretically, the prefactors γ, $\chi_{0}$, and *A* all depend on the renormalized electronic density of states $N^{*}=N/N_{0}$, or the related renormalized (density-of-states) effective mass $m^{*}=m/m_{0}∼N^{*}$, to first approximation as $\gamma ∼m^{*}$, $\chi_{0}∼m^{*}$, and $A∼{\left(m^{*}\right)}^{2}$. $N_{0}$ and $m_{0}$ are the free electron quantities. Indeed, in double-logarithmic plots of γ vs. $\chi_{0}$ (Sommerfeld-Wilson) and *A* vs. γ (Kadowaki-Woods), experimental data of a large number of heavy fermion compounds fall on universal lines, thereby confirming the theoretically expected universal ratios [28]. The scaling works close to perfectly if corrections due to different ground state degeneracies [29] and effects of dimensionality, electron density, and anisotropy [30] are taken into account.

More surprising, then, was the discovery that this very robust Fermi liquid behavior can nevertheless cease to exist. This can have multiple reasons, but the predominant and best investigated one is quantum criticality [4,25,31,32]. In the standard scenario for quantum criticality of itinerant fermion systems [33,34,35], a continuously vanishing Landau order parameter (typically of a density wave) governs the physical properties. Its effect on the electrical resistivity is expected to be modest because (i) the long-wavelength critical modes of the bosonic order parameter can only cause small-angle scattering, which does not degrade current efficiently, and (ii) critical density wave modes only scatter those areas on the Fermi surface effectively that are connected by the ordering wavevector. Fermions from the rest of the Fermi surface will short circuit these hot spots [36]. For itinerant ferromagnets, $\rho ∼T^{5/3}$ is theoretically predicted [4] and experimentally observed [37]. For itinerant antiferromagnets, this type of order-parameter quantum criticality should result in $\rho ∼T^{\epsilon }$ with 1≤ϵ≤1.5, depending on the amount of disorder [36]. Whereas this may be consistent with experiments on a few heavy fermion compounds, a strong dependence of ϵ with the degree of disorder has, to the best of our knowledge, not been reported. More importantly, for relatively weak disorder, the current is dominated by the contributions from the cold regions of the Fermi surface which stay as quasiparticles and the resistivity would have the $T^{2}$ dependence of a Fermi liquid [38].

**Figure 1:**
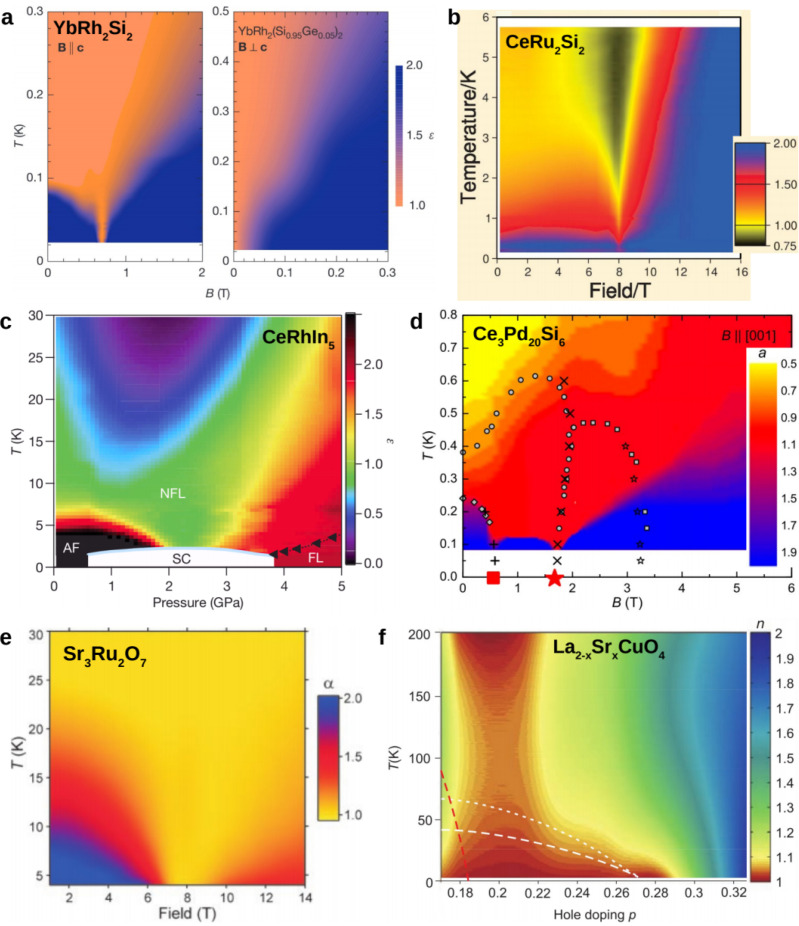

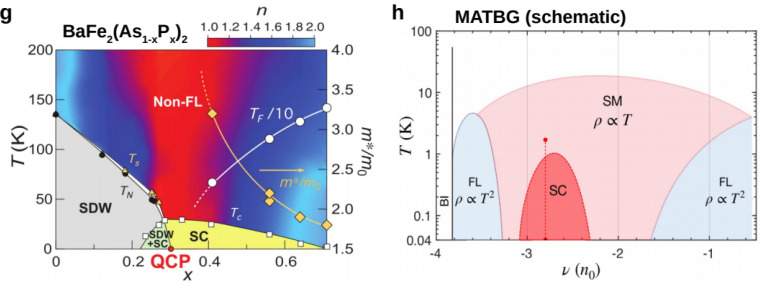
Color-coded phase diagrams featuring strange metal behavior in various materials platforms. (**a**) YbRh${}_{2}$Si${}_{2}$ (**left**) and YbRh${}_{2}$(Si${}_{0.95}$Ge${}_{0.05}$)${}_{2}$ (**right**), from [40]. (**b**) CeRu${}_{2}$Si${}_{2}$, from [41]. (**c**) CeRhIn${}_{5}$, from [42]. (**d**) Ce${}_{3}$Pd${}_{20}$Si${}_{6}$, from [43]. (**e**) SrRu${}_{3}$O${}_{7}$. Note that the temperature scale is cut at 4.5 K. At lower temperatures, deviations from linear behavior towards larger powers are observed; from [44]. (**f**) La${}_{2-x}$Sr${}_{x}$CuO${}_{4}$, from [45]. (**g**) BaFe${}_{2}$(As${}_{1-x}$P${}_{x}$)${}_{2}$, from [46]. (**h**) Magic-angle twisted bi-layer graphene, adapted from [47].

Instead, a number of heavy fermion compounds exhibit a linear-in-temperature electrical resistivity

(7) $$\rho =\rho_{0}^{\prime }+A^{\prime }T\;,$$

a dependence dubbed “strange metal” behavior from the early days of high-temperature superconductivity on [39]. In Figure 1a–d we show four examples, in the form of temperature–magnetic field (a,b,d) or temperature–pressure (c) phase diagrams with color codings that reflect the exponent ϵ of the temperature-dependent inelastic electrical resistivity, $\Delta \rho \propto T^{\epsilon }$, determined locally as ϵ=∂(lnΔρ)/∂(lnT). In all cases, fans of non-Fermi liquid behavior (ϵ≠2) appear to emerge from QCPs, with ϵ close to 1 in the center of the fan and extending to the lowest accessed temperatures (at least in a,c,d).

The most pronounced such behavior is found in YbRh${}_{2}$Si${}_{2}$ (Figure 1a, left). Below 65 mK, the system orders antiferromagnetically [48]. As magnetic field (applied along the crystallographic *c* axis) continuously suppresses the order to zero at 0.66 T [40], linear-in-temperature resistivity, with $A^{\prime }=1.8\;\mu \Omega$cm/K and $\rho_{0}^{\prime }=2.43\;\mu \Omega$cm, extends from about 15 K [48] down to the lowest reached temperature (below 25 mK) [40]. Recently, this range was further extended down to 5 mK, showing $A^{\prime }=1.17\;\mu \Omega$cm/K for a higher-quality single crystal ($\rho_{0}^{\prime }=1.23\;\mu \Omega$cm) [49], thus spanning in total 3.5 orders of magnitude in temperature. This happens in a background of Fermi liquid behavior away from the QCP. A linear-in-temperature resistivity is also seen in the substituted material YbRh${}_{2}$(Si${}_{0.95}$Ge${}_{0.05}$)${}_{2}$. Its residual resistivity is about five times larger than that of the stoichiometric compound. That this sizeably enhanced disorder does not change the power ϵ indicates that the order-parameter-fluctuation description of an itinerant antiferromagnetic quantum critical point [36] is not appropriate here. This point will be further discussed in Section 7.

For CeRu${}_{2}$Si${}_{2}$ (Figure 1b), the situation is somewhat more ambiguous. Linear-in-temperature resistivity does not cover the entire core region of the fan; both above 2 K and below 0.5 K, crossovers to other power laws can be seen [41]. In CeRhIn${}_{5}$ (Figure 1c), at the critical pressure of 2.35 GPa, linear-in-temperature resistivity extends from about 15 K down to 2.3 K, the maximum critical temperature of a dome of unconventional superconductivity [42]. That the formation of emergent phases such as unconventional superconductivity tends to be promoted by quantum critical fluctuations is, of course, of great interest in its own right even if, pragmatically, it can be seen as hindering the investigation of the strange metal state. Finally, Ce${}_{3}$Pd${}_{20}$Si${}_{6}$ exhibits two consecutive magnetic field-induced QCPs, with linear-in-temperature resistivity emerging from both [43]. Other heavy fermion systems show similar behavior, though color-coded phase diagrams may not have been produced. A prominent example is CeCoIn${}_{5}$. Its electrical resistivity was first broadly characterized as being linear-in-temperature below 20 K down to the superconducting transition temperature of 2.3 K [50]. Both magnetic field [51,52] and pressure [53] suppress the linear-in-temperature dependence and stabilize Fermi liquid behavior, in agreement with temperature over magnetic field scaling of the magnetic Grüneisen ratio indicating that a quantum critical point is situated at zero field [54]. Indeed, small Cd doping stabilizes an antiferromagnetic state [55].

In Figure 1e–h, we show resistivity-exponent color-coded phase diagrams of other classes of strongly correlated materials, a ruthenate, a cuprate, an iron pnictide, and a schematic phase diagram of MATBG. Extended regions of linear-in-temperature resistivity are also observed. Before we discuss this strange metal behavior in more detail in Section 5, we take a closer look at the Fermi liquid regions of the heavy fermion phase diagrams.

## 4. Fermi Liquid Behavior near Quantum Critical Points

**Figure 2:**
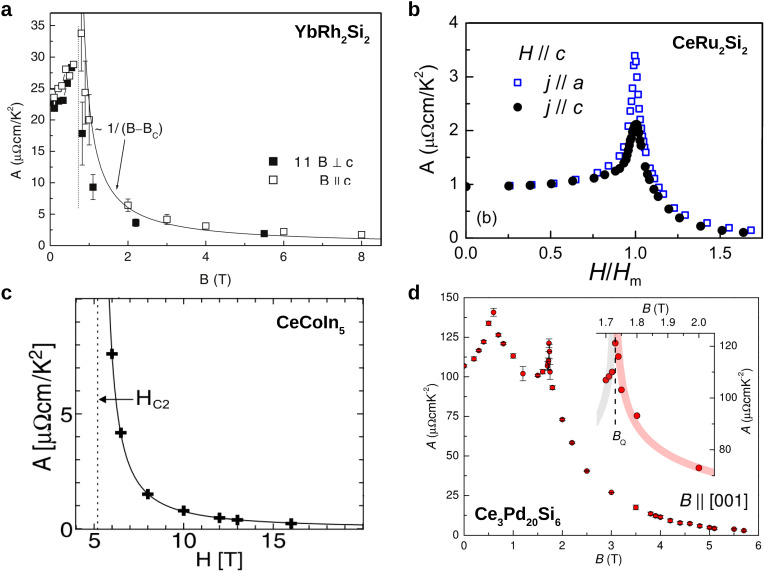
Variation of the *A* coefficient of the Fermi liquid form of the electrical resistivity, $\rho =\rho_{0}+AT^{2}$, across QCPs in various heavy fermion compounds. (**a**) YbRh${}_{2}$Si${}_{2}$, from [40]. (**b**) CeRu${}_{2}$Si${}_{2}$, from [56]. (**c**) CeCoIn${}_{5}$, from [51]. (**d**) Ce${}_{3}$Pd${}_{20}$Si${}_{6}$, from [43].

The low energy scales and associated low magnetic ordering temperatures typically found in heavy fermion compounds call for investigations of these materials at very low temperatures. Indeed, since early on, measurements down to dilution refrigerator temperatures have been the standard. Because scattering from phonons is strongly suppressed at such low temperatures, this is ideal to study non-Fermi liquid and Fermi liquid behavior alike. The phase diagrams in Figure 1a–d all feature Fermi liquid regions, at least on the paramagnetic side of the QCPs. The fan-like shape of the quantum critical regions dictates that the upper bound of the Fermi liquid regions shrinks upon approaching the QCP. Nevertheless, high-resolution electrical resistivity measurements still allow to extract the evolution of the Fermi liquid *A* coefficient upon approaching the QCP. In Figure 2 we show such dependencies for four different heavy fermion compounds. In all cases, the *A* coefficient is very strongly enhanced towards the QCP. In fact, within experimental uncertainty, the data are even consistent with a divergence of *A* at the QCP, as indicated by the power law fits, $A∼1/{\left(B-B_{c}\right)}^{a}$, with *a* close to 1, in Figure 2a,c,d, suggesting that the effective mass diverges at the QCP.

This finding challenges the classification of heavy fermion compounds into lighter and heavier versions, that has been so popular in the early days of heavy fermion studies and that had culminated in the celebrated Kadowaki–Woods and Sommerfeld–Wilson plots, with each heavy fermion compound represented by a single point. Which *A* (γ, $\chi_{0}$) value should now be used in these graphs? In [32] the use of lines instead of points was suggested, using the largest and smallest actually measured values (and not extrapolations beyond them) as end points. The question that remains is whether there is a “background” value, away from a quantum critical point, that is characteristic of a given compound. We will get back to this question in the next section.

## 5. Strange Metal Behavior and Planckian Dissipation

The occurrence of fans or, in some cases, differently shaped regions of linear-in-temperature resistivity in the phase diagrams of a broad range of correlated electron systems, as highlighted in Figure 1, raises the question whether a universal principle may be behind it. A frequently made argument is that linear-in-temperature resistivity is a natural consequence of the systems’ energy scales vanishing at a quantum critical point and thus temperature becoming the only relevant scale. However, both the experimental observation of power laws $\Delta \rho ∼T^{\epsilon }$ with ϵ≠1 in quantum critical heavy fermion compounds [57,58,59,60] and predictions from order-parameter-fluctuation theories of such laws [36] tell us that this argument cannot hold in general. We thus have to be more specific and ask whether for quantum critical systems that *do* exhibit linear-in-temperature resistivities and, apparently, require description beyond this order-parameter framework, a universal understanding can be achieved.

A direction that is attracting considerable attention [10,11,61] is to test whether the transport scattering rate 1/τ of such systems may be dictated by temperature via

(8) $$\frac{1}{\tau }=\alpha \frac{k_{B}T}{\hbar }$$

with α≈1. Should this be the case and τ be the only temperature-dependent quantity in the electrical resistivity, then a linear-in-temperature resistivity would follow naturally. Conceptually, this roots in the insight, gained from the study of models without quasiparticles [4,62,63,64,65], that a local equilibration time (after the action of a local perturbation) of any many-body quantum system cannot be faster than the Planckian time

(9) $$\tau_{P}=\frac{\hbar }{k_{B}T}$$

associated with the energy $k_{B}T$ via the Heisenberg uncertainty principle [65]. The question then is how to experimentally test this scenario. The simplest starting point is the Drude form for the electrical resistivity which, in the dc limit, reads

(10) $$\rho =\frac{m}{ne^{2}}\frac{1}{\tau }\;,$$

with a temperature-independent effective mass *m* and charge carrier concentration *n*, and (8) for the scattering rate 1/τ, leading to

(11) $$\rho =\alpha \frac{m}{ne^{2}}\frac{k_{B}T}{\hbar }\;.$$

Interpreting this as the inelastic part of the linear-in-temperature electrical resistivity (7), with $d\rho /dT=A^{\prime }$, one obtains

(12) $$\alpha =\frac{n}{m}\frac{e^{2}\hbar }{k_{B}}A^{\prime }$$

or, in convenient units format,

(13) $$\alpha =2.15\cdot \frac{n({\mathrm{nm}}^{-3})}{m/m_{0}}\cdot A^{\prime }\left(\mu \Omega \mathrm{cm}/K\right)\;,$$

where $m_{0}$ is the free electron mass. When this results in α≈1, the dissipation is said to be “Planckian”. Before looking at experiments, let’s contemplate this for a moment. Relation (12) is based on the simple Drude model, and combines properties of well defined quasiparticles (*n* and *m*) with a property that characterizes a non-Fermi liquid ($A^{\prime }$)—possibly one without quasiparticles—that is unlikely to follow the Drude model. Furthermore, as shown in Section 4, the Fermi liquid *A* coefficient, which is a measure of *m*, varies strongly with the distance to the QCP. Another defining property of at least some of these strange metals are Fermi surface jumps at the QCP (see Section 7). This adds a nontrivial temperature and tuning parameter dependence to *n*. One should thus bear in mind that choosing a simple Drude model as starting point holds numerous pitfalls. If still doing so, it is unclear which *m* and *n* value to use.

In [10], published quantum oscillation data, in part combined with results from density functional theory (DFT), were used to estimate *m* and *n* for a range of different materials, including also “bad metals” (see Section 6) and simple metals in the regime where their resistivity is linear-in-temperature due to scattering from phonons. As an example, for Sr${}_{3}$Ru${}_{2}$O${}_{7}$, de Haas–van Alphen (dHvA) data [66] measured at dilution refrigerator temperatures on the low-field side of the strange metal fan (Figure 1e) were used. Contributions from the different bands, assumed as strictly 2D, were summed up as

(14) $$\sigma =\tau \frac{e^{2}}{\hbar }\sum_{i}\frac{n_{i}}{m_{i}}\;,$$

i.e., a constant relaxation time was assumed for all bands. Then, the heavy bands with small carrier concentration play only a minor role. In this way, α=1.6 was obtained. The dHvA effective masses of all bands were found to be modest (at most $10m_{0}$) and essentially field-independent [66], even though the *A* coefficient increases by more than a factor of 8 on approaching the strange metal regime from the low field side [66]. The dHvA experiments may thus not have detected all mass enhancement [10,66]. As shown below, using a larger effective mass would reduce α.

**Figure 3:**
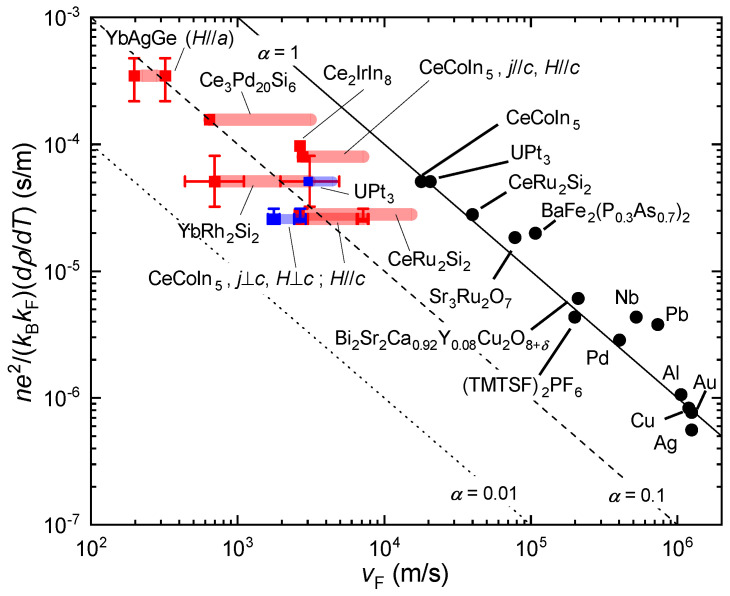
Planckian dissipation plot of [10] revisited. Double-logarithmic plot of Fermi velocity $v_{F}$ vs. $ne^{2}/\left(k_{B}k_{F}\right)\left(d\rho /dT\right)=\alpha /v_{F}$ with data from [10] (black points) and data of the heavy fermion compounds listed in Table 1 and analyzed here. The red squares result from the largest measured *A* coefficient (or γ value) for each compound near the strange metal regime, the shaded red lines from the published ranges of *A* coefficient (or γ value), and the error bars from uncertainties in the determination of the charge carrier concentration *n* and sometimes other parameters (see Table 1). The full, dashed, and dotted line represent α=1, 0.1, and 0.01, respectively.

Similar analyses were performed for the other materials [10] and we replot the results as black points in Figure 3. The *x* axis of this plot is the Fermi velocity $v_{F}$ which, for a 3D system, can be brought into the form

(15) $$v_{F}\left(m/s\right)=3.58\cdot 10^{5}\cdot \frac{\left[n{(\mathrm{nm}}^{-3}\right){]}^{1/3}}{m/m_{0}}\;.$$

The *y* axis is the inverse of $v_{F}$ multiplied by α (13) which, again for a 3D system, can be written as

(16) $$\frac{\alpha }{v_{F}}\left(s/m\right)=6.01\cdot 10^{-6}\cdot A^{\prime }\left(\mu \Omega \mathrm{cm}/K\right)\cdot \left[n{(\mathrm{nm}}^{-3}\right){]}^{2/3}\;.$$

To further assess how the results for α depend on the choice of the quasiparticle parameters *m* and *n*, we here take a different approach. Instead of quantum oscillation data, we use global (effective) properties, namely, the *A* coefficient and the Hall coefficient $R_{H}$, and estimate α for a number of strange metal heavy fermion compounds. Because of the extreme mass renormalizations observed in this class of materials (see Section 4), it is particularly well suited for this test. Combining

(17) $$\frac{m}{m_{0}}\cdot n^{1/3}=\frac{\gamma_{\mathrm{mole}-f.u.}}{V_{f.u.}}\frac{3\hbar^{2}}{N_{A}m_{0}k_{B}^{2}{\left(3\pi^{2}\right)}^{1/3}}$$

with the Kadowaki–Woods ratio $A/\gamma^{2}=10^{-5}\;\mu \Omega$cm(mole K/mJ)${}^{2}$, which is known to be very well obeyed in heavy fermion compounds [28], we obtain

(18) $$\frac{m}{m_{0}}\cdot {\left[n\left({\mathrm{nm}}^{-3}\right)\right]}^{1/3}=3.26\cdot 10^{4}\frac{\sqrt{A(\mu \Omega \mathrm{cm}/K^{2})}}{V_{f.u.}\left({Å}^{3}\right)}\;.$$

The rationale for using *A* instead of γ is that precise resistivity measurements are most abundant in the literature (also under challenging conditions such as high pressure and magnetic field) and that the resistivity is much less sensitive to extra contributions from phase transitions than the specific heat. In addition, and unlike γ, the *A* coefficient picks up effective mass anisotropies, which further improves our analysis. In all cases where reliable γ values were available [43,67,68,69], the agreement with our *A* coefficient γ was satisfactory.

A note is due on the determination of the charge carrier concentration *n*. It is commonly extracted from the Hall coefficient $R_{H}$, using the simple one-band relation $R_{H}=1/ne$. Heavy fermion compounds are typically multiband systems, and thus compensation effects from electron and hole contributions can occur [70]. To limit the effect of anomalous Hall contributions, low-temperature data should be used [71]. Quantum oscillation experiments can determine the carrier concentration of single bands. However, heavy bands are hard to detect and it is unclear how to sum up contributions from different bands. An alternative is to determine *n* via the superfluid density [72], as was done previously [49,73], using the relation (in cgs units)

(19) $$n={\left(\frac{\xi_{0}\cdot T_{c}\cdot \gamma }{7.95\cdot 10^{-24}}\right)}^{3/2}\;,$$

where $\xi_{0}$ is the superconducting coherence length, $T_{c}$ is the superconducting transition temperature, and γ is the normal-state Sommerfeld coefficient, which can be rewritten as

(20) $$n\left({\mathrm{nm}}^{-3}\right)=3020\cdot {\left(\frac{\xi_{0}\left(\mathrm{nm}\right)\cdot T_{c}\left(K\right)\cdot \gamma \left({\mathrm{Jmol}}^{-1}K^{-2}\right)}{V_{f.u.}\left({Å}^{3}\right)}\right)}^{3/2}\;.$$

This may be used as a lower bound of the carrier concentration in the normal state.

Table 1 lists the materials we inspected, with their *A* coefficients (or, when unavailable, γ), the best estimate of the charge carrier concentration *n* following the above discussion (see Table 2 for details), and the strange metal $A^{\prime }$ coefficient. $m/m_{0}$ as calculated via (18), or (17), is also listed.

**Table 1:** Parameters used for Figure 3 and Figure 4. The red (or blue) square represents the largest *A* coefficient (measured closest to the QCP), the shaded red (or blue) lines the range of *A* coefficient measured upon moving away from the QCP. The Sommerfeld coefficient γ is estimated from *A* via the Kadowaki–Wood ratio, unless *A* data are unavailable. The charge carrier concentrations *n* and their error bars (where applicable) are taken from Table 2. For CeCoIn${}_{5}$, several values are listed because the *A* coefficient is different for in-plane ($H_{a}$) and out-of-plane ($H_{c}$) field, and the $A^{\prime }$ coefficient is different for in-plane ($j_{a}$) and out-of-plane ($j_{c}$) currents. For YbAgGe, the $A^{\prime }$ coefficient changes with field; the two extreme $A^{\prime }$ values are denoted by the two red squares. For CeCoIn${}_{5}$ (j⊥c), Figure 3 shows the range $A^{\prime }=\left(0.8\pm 0.2\right)\;\mu \Omega$cm/K from [74]. Data for Ce${}_{3}$Pd${}_{20}$Si${}_{6}$ refer to the second QCP (near 2 T, see Figure 1d) because for the lower field QCP no full data set on single crystals is published [43,75].

Compound	*A* (μΩcm/K${}^{2}$)	γ (J/molK${}^{2}$)	$m/m_{0}$	*n* (nm${}^{-3}$)	$A^{\prime }$ (μΩcm/K)
Ce${}_{2}$IrIn${}_{8}$	–	0.65 [76]	183	2.5	8.8 [76]
Ce${}_{3}$Pd${}_{20}$Si${}_{6}$	5–120 [43]	0.707–3.46	136–665	1.7	18.3 [43]
CeCoIn${}_{5}$ ($j_{a},H_{a}$)	12.4–28.3 [67]	1.11–1.68	310–470	12.4	0.8 [77]
CeCoIn${}_{5}$ ($j_{a},H_{c}$)	1.72–11.5 [67]	0.414–1.07	116–300	12.4	0.8 [77]
CeCoIn${}_{5}$ ($j_{c},H_{c}$)	1.72–11.5 [67]	0.414–1.07	116–300	12.4	2.475 [77]
CeRu${}_{2}$Si${}_{2}$	0.1–3.4 [56]	0.1–0.583	53–310	11.6	0.91 [41]
UPt${}_{3}$	–	0.425–0.625 [78]	223–329	21.4	1.1 [10]
YbAgGe (H//a)	–	0.87–1.4 [79]	1300–2100	1.6	27–59 [80]
YbRh${}_{2}$Si${}_{2}$	1.7–33.8 [68]	0.41–1.85	250–1100	10	1.83 [68]

All these data are then included in Figure 3 in the following way. The $v_{F}$ (15) and $\alpha /v_{F}$ (16) value resulting from the largest measured *A* coefficient (or γ value) for each compound is shown as red square. The shaded red lines represent the published ranges of *A* coefficient (or γ value). The error bars represent uncertainties in the determination of the charge carrier concentration (see Table 1). Lines for α=1, 0.1, and 0.01 are also shown. It is clear that none of the shaded red lines overlaps with the α=1 line. The discrepancy with the points extracted from quantum oscillation experiments [10] is quite striking.

**Figure 4:**
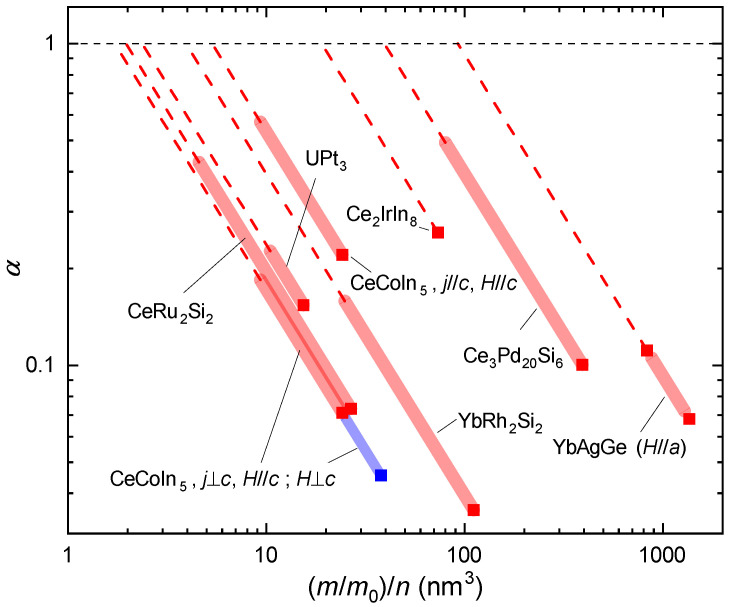
No Planckian dissipation from heavy quasiparticles in heavy fermion compounds. Double-logarithmic plot of α vs. $(m/m_{0})/n$ for various strange metal heavy fermion compounds, as given in Table 1. Red squares and shaded lines have the same meaning as in Figure 3. The dashed lines are to help reading off the values of $(m/m_{0})/n$ for which the linear-in-temperature electrical resistivity in these compounds could be governed by Planckian dissipation. Note that in all cases the “Planckian dissipation” effective masses obtained in this way are sizeably smaller than even the smallest values experimentally accessed by tuning the systems away from the strange metal regime (top end of full shaded lines).

In Figure 4 we present these results in a different form, as α vs. $(m/m_{0})/n$. The red squares and red shaded lines have the same meaning as in Figure 3. The dashed lines are extrapolations of the shaded lines to α=1. We can thus directly read off the values of $(m/m_{0})/n$ for which a given compound would, in this simple framework, be a Planckian scatterer. In all cases, this is for effective masses significantly smaller than even the smallest measured ones in the Fermi liquid regime.

What are the implications of this finding? We first comment on the discrepancy with the results from [10]. Apparently, averaging the contributions from different bands detected in quantum oscillation experiments via (14) leads to sizeably larger Fermi velocities (sizeably smaller effective masses) than our *A* coefficient approach. In heavy fermion compounds, a coherent heavy fermion state forms at low temperatures, and the Fermi liquid *A* coefficient is known to be a pertinent measure thereof. It is thus either the use of (14) that should be reconsidered or the reliance in quantum oscillation experiments to detect the heaviest quasiparticles. Clearly, if dissipation in strange metal heavy fermion compounds is to be Planckian, this would hold only for the very weakly renormalized quasiparticles, as argued for in [88]. To us, this is a rather puzzling result as heavy fermion bands get successively renormalized with decreasing temperature and thus one would have expected that the “background” to effects of quantum critical fluctuations already contains a sizeable non-critical Kondo renormalization.

## 6. Strange Metal Behavior and the Mott–Ioffe–Regel Limit

In a number of strongly correlated electron systems, including quasi-2D conductors such as the high-$T_{c}$ cuprates but also 3D transition metal oxides and alkali-doped fullerides, linear-in-temperature resistivity is observed beyond the Mott–Ioffe–Regel (MIR) limit [89,90]. At this limit, the electron mean free path approaches certain microscopic length scales such as the interatomic spacing or the wavelength $2\pi /k_{F}$ [65,91,92,93,94]. Semiclassical transport of long-lived quasiparticles might then, at least naively, be expected not to exist and the resistivity should saturate, in 3D systems of interest to us here to

(21) $$\rho_{\mathrm{MIR}}=\frac{h}{e^{2}}\cdot L\;,$$

where *L* is the relevant microscopic length scale. Using the Drude resistivity (10) with the Fermi velocity $v_{F}=\hbar k_{F}/m$, the Fermi wave vector $k_{F}={\left(3\pi^{2}n\right)}^{1/3}$, and the mean free path $\ell =\tau v_{F}$ one obtains

(22) $$\rho =\frac{h}{e^{2}}\cdot \frac{3\pi }{2}\frac{1}{k_{F}^{2}\ell }=\frac{h}{e^{2}}\cdot L\cdot C\;,$$

where the value of the constant *C* depends on details of the electronic and crystal structure. Assuming C=1, one gets

(23) $$\rho_{\mathrm{MIR}}\left(\mu \Omega \mathrm{cm}\right)=258\cdot L\left(Å\right)\;,$$

In heavy fermion compounds, linear-in-temperature resistivities are limited to low temperatures (Figure 1a–d) and the $A^{\prime }$ coefficients (Table 1) typically result in inelastic resistivities of the order of 10 μΩcm at the upper bound of the linear regime. This is well below the MIR limit. For instance, for YbRh${}_{2}$Si${}_{2}$, using the lattice parameters a=4.007 Å and c=9.858 Å [48] for *L* in (23) gives $\rho_{\mathrm{MIR}}\approx 1000\;\mu \Omega$cm and ≈2500μΩcm, respectively, much larger than even the total resistivity at 15 K (which is about 30 μΩcm for YbRh${}_{2}$Si${}_{2}$ [48]), the upper bound of linear-in-temperature resistivity for that compound. In this case, a confusion with a linear-in-temperature resistivity due to electron-phonon scattering [65,95] can be safely ruled out.

## 7. Strange Metal Behavior and Fermi Surface Jumps

In Section 5, a simple Drude form was used for the electrical resistivity and all temperature dependence was attributed to the scattering rate. Then, the question was asked which quasiparticles (with which m/n) to take if the scattering were to be Planckian. The answer was that this would have to be very weakly interacting quasiparticles, certainly not the ones close to the QCP from which the strange metal behavior emerges. Here we address another phenomenon that may challenge a Planckian scattering rate picture: Fermi surface jumps across these QCPs.

**Figure 5:**
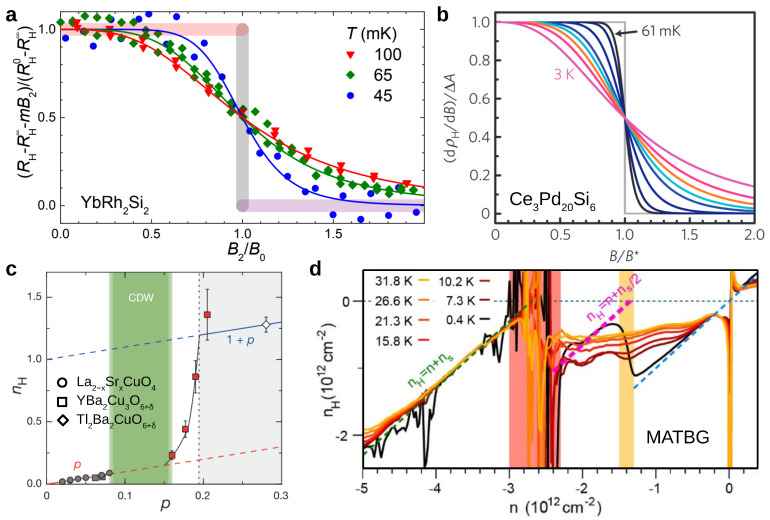
Fermi surface jumps as evidenced by Hall effect measurements in several strange metals. (**a**) YbRh${}_{2}$Si${}_{2}$, from [32,96]. (**b**) Ce${}_{3}$Pd${}_{20}$Si${}_{6}$, from [75]. (**c**) Substitution series of three high-$T_{c}$ cuprates, from [99]. (**d**) MATBG, from [100].

This phenomenon was first detected by Hall effect measurements on YbRh${}_{2}$Si${}_{2}$ [71,96] (Figure 5a). Let us first recapitulate the experimental evidence for a Fermi surface jump across a QCP, as put forward in these works. Hall coefficient $R_{H}$ (or Hall resistivity $\rho_{H}$) isotherms are measured as function of a tuning parameter δ (in case of YbRh${}_{2}$Si${}_{2}$ the magnetic field) across the QCP. A phenomenological crossover function, $R_{H}^{\infty }-\left(R_{H}^{\infty }-R_{H}^{0}\right)/\left[1+{\left(\delta /\delta_{0}\right)}^{p}\right]$[71], is fitted to $R_{H}\left(\delta \right)$ [or to $d\rho_{H}/dB\left(\delta \right)$] and its full width at half maximum (FWHM) is determined as a reliable measure of the crossover width. Only if this width extrapolates to zero in the zero-temperature limit a Hall coefficient jump is established. Of course, the jump size must remain finite in the zero temperature limit. To identify a Fermi surface jump, other origins of Hall effect changes must be ruled out, for instance anomalous Hall contributions from abrupt magnetization changes at a metamagnetic/first order transition [97]. All this was done for YbRh${}_{2}$Si${}_{2}$ [71,96]. For Ce${}_{3}$Pd${}_{20}$Si${}_{6}$, using a very similar procedure, two Fermi surface jumps were found at the two consecutive QCPs (Figure 1d) [43,75]. The crossover at the first QCP [75] is shown in Figure 5b. It is also important to remind oneself that no Fermi surface discontinuity is expected at a conventional antiferromagnetic QCP as described by the spin-density-wave/order-parameter scenario [6]. Band folding of the (even at T=0) continuously onsetting order parameter can in that case only lead to a continuously varying Hall coefficient, as seen for instance in the itinerant antiferromagnet Cr upon the suppression of the order by doping or pressure (see [98] for more details and the original references).

These jumps are understood as defining signatures of a Kondo destruction QCP, first proposed theoretically [5,6,101] in conjunction with inelastic neutron scattering experiments on CeCu${}_{5.9}$Au${}_{0.1}$ [9]. At such a QCP, the heavy quasiparticles, composites with *f* and conduction electron components, disintegrate. The Fermi surface jumps because the local moment, which is part of the Fermi surface in the paramagnetic Kondo coherent ground state [102], drops out as the *f* electrons localize. As such, Kondo destruction QCPs are sometimes referred to as *f*-orbital selective Mott transitions. More recently, THz time-domain transmission experiments on YbRh${}_{2}$Si${}_{2}$ thin films grown by molecular beam epitaxy revealed dynamical scaling of the optical conductivity [103]. This shows that the charge carriers are an integral part of the quantum criticality, and should not be seen as a conserved quantity that merely undergo strong scattering (as in order-parameter-fluctuation descriptions with intact quasiparticles). We also note that a Drude description of the optical conductivity fails rather drastically in the quantum critical regime [103]. It is thus unclear how this physics could be captured by the simple Planckian scattering approach described above.

Interestingly, Hall effect experiments in other strange metal platforms also hint at Fermi surface reconstructions. Two examples are included in Figure 5: a series of substituted high-$T_{c}$ cuprates [99] (panel c) and MATBG as function of the total charge density induced by the gate [100] (panel d). Evidence for related physics has also been found in the pnictides [104]. The physics here appears to be related to the presence of *d* orbitals with a different degree of localization, with one of them undergoing a Mott transition, such as described by multi-orbital Hubbard models [105,106]. It may well be that Fermi surface jumps are an integral part of strange metal physics, and should be included as a starting point in its description.

## 8. Summary and Outlook

We have revisited the question whether the strange metal behavior encountered in numerous strongly correlated electron materials may be the result of Planckian dissipation. For this purpose, we have examined strange metal heavy fermion compounds. Their temperature–tuning parameter phase diagrams are particularly simple: Fans of strange metal behavior emerge from quantum critical points, in a Fermi liquid background. This, together with the extreme mass renormalizations found in these materials, makes them a particularly well-suited testbed.

As done previously, we use the Drude form of the electrical conductivity as a starting point, but complementary to a previous approach based on quantum oscillation data, we here rely on the Fermi liquid *A* coefficient as precise measure of the quasiparticle renormalization. We find that for any of the measured *A* coefficients, the slope of the linear-in-temperature strange metal resistivity $A^{\prime }$ is much smaller than the value expected from Planckian dissipation. We also propose a new plot that allows to read off the ratio of effective mass to carrier concentration that one would have to attribute to the quasiparticles for their scattering to be Planckian. It corresponds to very modest effective masses. While this could be something like a smooth background to quantum critical phenomena, the fact that the strange metal regime occurs entirely below the temperature for the initial onset of the dynamical Kondo correlations suggests that this background should already incorporate the non-critical Kondo correlations and thus correspond to a relatively heavy mass.

We have also pointed out that several heavy fermion compounds exhibit Fermi surface jumps across strange metal quantum critical points and that this challenges the Drude picture underlying the Planckian analysis. Indications for such jumps are also seen in other platforms and may thus be a common feature of strange metals. Further careful studies that evidence a sharp Fermi surface change in the zero temperature limit, such as providing for some of the heavy fermion compounds, are called for. On the theoretical side, approaches that discuss the electrical resistivity as an entity and do not single out a scattering rate as the only origin of strangeness, are needed.

## Figures and Tables

**Table 2:** Charge carrier concentrations (in nm${}^{-3}$) determined as follows: (i) $n_{\mathrm{sc}}$ from the superconducting coherence length $\xi_{0}$, the superconducting transition temperature $T_{c}$, and the normal-state Sommerfeld coefficient γ, all in zero field, via (20); (ii) $n_{H}$ from the Hall coefficient at the lowest temperatures, where anomalous contributions are minimal, via $R_{H}=1/ne$; (iii) $n_{\mathrm{qo}}$ from quantum oscillation experiments reviewed in [10], by summing up the carrier concentrations from all detected bands. For CeCoIn${}_{5}$, the γ coefficient is taken at 2.5 K, without taking into account the logarithmic divergence. The error bar in *n* used for CeCoIn${}_{5}$ (j⊥c) in Figure 3 reflects the range of the parameters given in [74]. YbRh${}_{2}$Si${}_{2}$ is close to being a compensated metal, resulting in a strong sensitivity of *n* to small differences in the residual resistivity. The largest reported $R_{H}$ value, which corresponds to $n_{H}=26.0$[71], has the lowest compensation and is thus most accurate. Nevertheless, the $R_{H}$ value of LuRh${}_{2}$Si${}_{2}$ is even larger, corresponding to $n_{H}=11.6$ nm${}^{-3}$[70], suggesting that there is still some degree of compensation in the sample of [71]. We list the average of both values, 18.8 nm${}^{-3}$, as best $n_{H}$ estimate. For the plots, we use the approximate average of $n_{\mathrm{sc}}$ and $n_{H}$, i.e., 10 nm${}^{-3}$, with an asymmetric error bar $\delta n_{+}=10$ nm${}^{-3}$ and $\delta n_{-}=-5$ nm${}^{-3}$ (see Table 1). Similar compensation effects are also encountered in UPt${}_{3}$[81]. Bold fonts indicate the values used for the α estimates (see Table 1).

Compound	$\xi_{0}$ (nm)	$T_{c}$ (K)	γ (J/molK${}^{2}$)	$n_{\mathrm{sc}}$	$n_{H}$	$n_{\mathrm{qo}}$
Ce${}_{2}$IrIn${}_{8}$	-	-	-	-	**2.5** [82]	-
Ce${}_{3}$Pd${}_{20}$Si${}_{6}$	-	-	-	-	**1.7** [43]	-
CeCoIn${}_{5}$	5.6 [83]	2.3 [50]	290 [50]	10.8	10.1 [84]–12.5 [74]	**12.4** [10]
CeRu${}_{2}$Si${}_{2}$	-	-	-	-	3.1 [41]–7.8 [85]	**11.6** [10]
UPt${}_{3}$	12 [86]	0.52 [86]	0.43 [78]	22.4	9 [85]	**21.4** [10]
YbAgGe	-	-	-	-	**1.6** [87]	-
YbRh${}_{2}$Si${}_{2}$	97 [49]	0.0079 [49]	1.42 [49]	4.86	18.8 [70,71]	-

## Data Availability

No new data were created in this study. Data sharing is thus not applicable to this article.
